# First report of the *aac(6’)-Ib-cr* gene in *Providencia stuartii* isolates in Brazil

**DOI:** 10.1590/0037-8682-0524-2019

**Published:** 2020-11-13

**Authors:** Sivoneide Maria da Silva, Bárbara de Azevedo Ramos, Ana Vitória Araújo Lima, Rafael Artur Cavalcanti Queiroz de Sá, Jailton Lobo da Costa Lima, Maria Amélia Vieira Maciel, Patrícia Maria Guedes Paiva, Márcia Vanusa da Silva, Maria Tereza dos Santos Correia, Maria Betânia Melo de Oliveira

**Affiliations:** 1 Universidade Federal de Pernambuco, Centro de Biociências, Departamento de Bioquímica, Recife, PE, Brasil.; 2 Universidade Federal de Pernambuco, Centro de Ciências Médicas, Departamento de Medicina Tropical, Recife, PE, Brasil.

**Keywords:** Antimicrobial agents, Enterobacteriaceae, MDR genes, Providencia stuartii

## Abstract

**INTRODUCTION:**

The *aac(6’)-Ib-cr* and *bla*_KPC_ genes are spreading among *Enterobacteriaceae* species, including *Providencia stuartii*, in some countries of world.

**METHODS:**

These genes were investigated in 28 *P. stuartii* isolates from a public hospital in Recife, Pernambuco, Brazil, by PCR and sequencing.

**RESULTS:**

The *aac(6’)-Ib-cr* gene was detected in 16 resistant isolates, and the *bla*_KPC_ gene was seen in 14.

**CONCLUSIONS:**

The presence of these genes in *P. stuartii* multi- and extensively drug-resistant isolates indicates that the resistance arsenal of this species is increasing, thus limiting the therapeutic options.

*Providencia stuartii* belongs to the *Enterobacteriaceae* family and is commonly associated with urinary tract infections. However, it can cause other infections such as diarrhea, pneumonia, and septicemia^1^ ^-^ ^3^. One of the main causes of the pathogenicity of this species is its intrinsic resistance to various antimicrobials including some β-lactams and aminoglycosides, as well as tigecycline, colistin, and polymyxin B, which are used when resistance to carbapenems is present^4^. In addition to its intrinsic resistance, *P. stuartii* may acquire genes that code for different enzymes, such as the *Klebsiella Pneumoniae* carbapenemase^5^.

Some β-lactam antimicrobial resistance genes, such as *bla*_KPC_ and *bla*_OXA_, have been identified in *P. stuartii* in Brazil^3^ ^-^ ^6^However, there are no reports of genes encoding aminoglycoside-modifying enzymes (AMEs), such as aminoglycoside acetyltransferases (AACs). These enzymes may alter the activity of this class of antimicrobials. In addition, the aminoglycoside 6′-N-acetyltransferase type Ib variant of the enzyme (AAC[6′]-Ib) has acquired the ability to modify fluoroquinolones, without significantly altering its activity against aminoglycosides. This is the first report describing the *aac(6’)-Ib-cr* gene in *P. stuartii* isolates in Brazil, as well as confirming the presence and dissemination of the *bla*_KPC_ gene in this species and reporting on the genetic diversity in isolates obtained from a public hospital in Recife, Pernambuco, Brazil.

A total of 28 isolates from different infection sites and different sectors of a public hospital in Recife, Pernambuco, Brazil, were collected between June 2017 and April 2018 (Figure 1). The samples were stored in glycerol (15%) at -80°C and in mineral oil at room temperature. For laboratory analysis, they were cultured in brain heart infusion broth (BHI) at 37°C for 24 h. This study was approved by the *Comitê de Ética em Pesquisa* of the *Universidade Federal de Pernambuco*, Brazil (Ref. No. 2.581.723). The identification of isolates and determination of minimum inhibitory concentrations (MICs) were performed according to the Clinical and Laboratory Standards Institute (CLSI)^7^ guidelines using an automated Vitek 2 Compact system (bioMérieux, Marcy-l’Étoile, France) using 11 antimicrobials (Table 1). Taxonomic confirmation of isolates was performed using matrix-assisted laser desorption ionization time-of-flight (MALDI TOF) mass spectrometry (MS) in a MALDI-TOF Autoflex III Mass Spectrometer (Bruker Daltonics, Billerica, MA, USA). The mass spectra obtained were compared with the MALDI Biotyper version 3.1 database.

**FIGURE 1:**
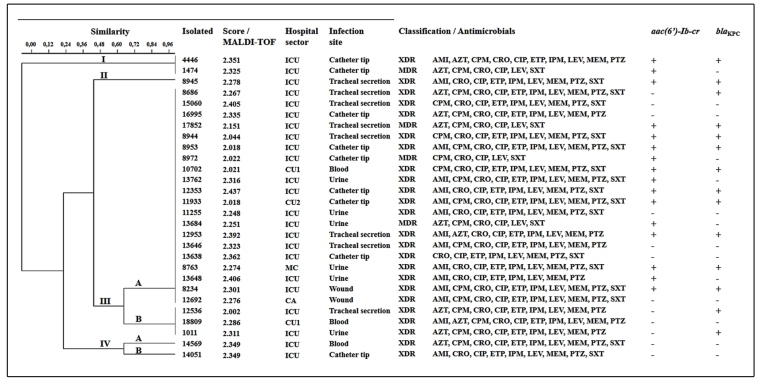
Phenotypic and genotypic characterization of the *Providencia stuartii* isolates. **AMI:** amikacin; **AZT:** aztreonam; Absent gene (-); **CA:** cardiology; **CPM:** cefepime; **CRO:** ceftriaxone; **CIP:** ciprofloxacin; **CU:** coronary unit; **ETP:** ertapenem; **XDR:** extensively drug-resistant; **ICU:** intensive care unit; **IPM:** imipenem; **LEV:** levofloxacin; **MC:** medical clinic; **MEM:** meropenem; **MDR:** multidrug-resistant; **PTZ:** piperacillin-tazobactam; Present gene (+); **SXT:** trimethoprim-sulfamethoxazole.

**TABLE 1:** Antimicrobial resistance profile of the *Providencia stuartii* isolates investigated.

Antimicrobials	MIC range (μg/mL)	Resistance (n = 28)
Amikacin	< 8 - > 32	15
Aztreonam	< 2 - > 16	11
Cefepime	< 1 - > 16	20
Ceftriaxone	≥ 16 - > 32	28
Ciprofloxacin	> 2	28
Ertapenem	< 0.25 - > 4	24
Imipenem	< 1 - > 8	24
Levofloxacin	> 4	28
Meropenem	< 1 - > 8	24
Piperacillin-Tazobactam	< 4 - > 64	24
Trimethoprim-Sulfamethoxazole	< 1 - > 4	20

**MIC:** minimum inhibitory concentration.

The *aac(6’)-Ib-cr* and *bla*_KPC_ genes were detected by polymerase chain reaction (PCR) using specific primer pairs and annealing temperatures^8^ ^-^ ^9^. Amplicons were evaluated using 1.2% agarose gel electrophoresis and 100 bp Ladder DNA marker (Invitrogen, Carlsbad, CA, USA). Subsequently, they were purified following the protocol given in the PureLink purification kit (Invitrogen) and sequenced on an ABI 3100 DNA automated apparatus. Data obtained by sequencing were analyzed and deposited in the Genbank database of the National Center for Biotechnology Information (NCBI), which provided the respective access numbers: MN371229 and MN371230. The enterobacterial repetitive intergenic consensus-PCR (ERIC-PCR) technique was performed to analyze the genetic diversity of the isolates. ERIC-PCR reactions were carried out and interpreted according to Duan et al.,^10^ as were the parameters for amplification. Amplicons were stained with Blue Green dye (LGC Biotechnology, SP, BR) and subjected to 1.5% agarose gel electrophoresis and visualized under ultraviolet light and photo-documented for later analysis of the clonal profiles. DARwin version 5.0 software was used to generate a dendrogram.

The isolates were confirmed as *P. stuartii* by MALDI-TOF MS, with scores between 2.002 and 2.437, indicating high similarity with this species. All isolates were resistant to ceftriaxone, ciprofloxacin, and levofloxacin. Twenty-four isolates were resistant to ertapenem, imipenem, meropenem, and piperacillin-tazobactam, and 20 were resistant to cefepime and trimethoprim-sulfamethoxazole. For amikacin and aztreonam, 15 and 11 resistant isolates were observed, respectively (Table 1). Based on the resistance profile, four isolates were characterized as multidrug-resistant (MDR) and the others were extensively drug-resistant (XDR). The *aac(6')-Ib-cr* gene was detected in 16 isolates, and the *bla*_KPC_ gene was seen in 14. Among the isolates, 11 were positive for both genes. However, 9 isolates did not contain the investigated genes (Figure 1).

In the present study, all isolates were confirmed by MALDI-TOF technique. The mass spectra obtained were compared with the reference spectra stored in databases using specific software. This technique is generally applied to identify a variety of microorganisms, especially those of clinical origin. Compared with other identification techniques (phenotypic characterization and *16S rDNA* gene sequencing), MALDI-TOF is used more frequently due to its low cost and high reliability^11^. In clinical practice, rapid and accurate pathogen identification is essential for adequate antimicrobial therapy.

All *P. stuartii* isolates were resistant to fluoroquinolone drugs (ciprofloxacin and levofloxacin). Regarding the β-lactam group, there was a significant proportion showing resistance to carbapenems (ertapenem, imipenem, and meropenem) and β-lactamase inhibitors (tazobactam), indicating low effectiveness of these antimicrobials on the *P. stuartii* isolates. Overall, amikacin and aztreonam were the antimicrobials to which the isolates presented the lowest resistance. Since the intrinsic resistance of this species is already recognized in the literature for aminoglycosides (except for amikacin), as well as for tigecycline, polymyxin B, or polymyxin E (colistin), used when there is resistance to carbapenems^7^, the combination of amikacin and aztreonam can be considered as a therapeutic option. The *Agência Nacional de Vigilância Sanitária* (ANVISA) in Brazil^12^ recommends a combination of aminoglycosides and β-lactams in the treatment of infections caused by MDR enterobacteria.

The resistance profiles of some of the isolates may be justified by the presence of the *aac(6')-Ib-cr* gene. This gene inactivates aminoglycosides and can confer resistance to some fluorquinolones^8^. Recently, Scavuzzi et al.^13^ reported the presence of the *aac(6')-Ib-cr* gene in *Klebsiella pneumoniae* samples from the city of Recife-PE. Our data show a probable spread of this gene among species of the *Enterobacteriaceae* family in public hospitals in Recife. Also, the *bla*_KPC_ gene has the potential to inactivate all β-lactams^14^. Thus, resistance to these antimicrobials may be due to the expression of this gene. Investigating the spread of *bla*_KPC_ in states from Brazil, Tavares et al.^5^ observed the presence of this gene in four *P. stuartii* isolates and highlighted the importance of the immediate recognition of this species as a carrier of this gene. Later, Aires et al.^3^ reported, in a hospital in Recife, an isolate of this species with *bla*_KPC_. Our study reinforces the spread of this gene in *P. stuartii* with a significantly higher number of isolates than previous studies and demonstrates the need for urgent measures to control infections. Enzyme production from plasmid or chromosomal genes represents the main resistance mechanism in MDR and XDR bacteria. However, other mechanisms, such as a low permeability of the outer membrane, which limits antimicrobial passage, changes in the efflux pumps, which removes the drug into the extracellular environment, and modifications to antimicrobial target proteins, also contribute to bacterial survival^14^. Thus, isolates that did not have these genes may employ other resistance mechanisms.

Antimicrobial resistance has significantly increased in recent years. It has led to the emergence of highly virulent strains such as carbapenem-resistant *Enterobacteriaceae*. Based on data presented by the World Health Organization^15^, infections caused by these bacteria are among the leading causes of morbidity and mortality in the world.

After verifying the resistance profile, this study investigated the clonal profile among *P. stuartii* isolates by ERIC-PCR, which detected four molecular profiles. Profile I included two clones, while profile II had only one. Profile III was divided into two subgroups: IIIA, with 20 clones, and IIIB, with only three. Profile IV was divided into subgroups: IVA and IVB, each with by one isolate (Figure 1). These data revealed the clonal dissemination of most isolates in different sectors of the studied hospital and demonstrated the need for more effective infection control measures.

The results show that the therapeutic options for *P. stuartii* are becoming increasingly limited. In addition to its natural resistance to various antimicrobials, including some advanced therapeutic options^4^ ^-^ ^7^, this species has demonstrated the potential to acquire other resistance genes. Routine epidemiological studies may help guide the synthesis of new drugs and the choice of appropriate treatments.
